# The American Dental Association

**Published:** 1878-09

**Authors:** 


AMERICAN JOURNAL

OF

DENTAL SCIENCE.

Vol. XII. THIRD SERIES—SEPTEMBER, 1878. No. 5.

### ARTICLE I.

### *The American Dental Association.*

The eighteenth annual session of the American Dental Association was held at Grant's Hall, Niagara Falls, commencing August 6th, the President, Dr. F. H. Rehwinkle, of Chillicothe, O., in the chair, and Dr. M. S. Dean, of Chicago, Recording Secretary. The Association was called to order at ten o'clock, and the proceedings opened with prayer by Dr. W. H. Morgan, of Nashville, Tenn.

Following the calling of the roll of qualified delegates by the Treasurer, Dr. W. H. Goddard, of Louisville, Ky., the minutes of the final session of the last annual meeting were read and approved.

Dr. Thomas Fillebrown, of Portland, Me., Chairman of the Committee on Programme, reported the following Order of Business:

Organization of the meeting, 10 A. M.

Calling the roll of qualified members.

Reading of the minutes, and action thereon.

Meeting of the Executive Committee, filling of vacancies therein, examination of credentials, and payment of dues, 8 A. M.

Reports of officers and Special Committees.

The reading and consideration of the stated annual reports from the Standing Committees, together with volunteer papers, upon the same subjects, in their consecutive order.

Report of Executive Committee.

Balloting for the place of next annual meeting.

Election of officers and three members of Executive Committee.

Appointment of Standing Committees.

Instructions to the Permanent Committees.

Unfinished, new, and miscellaneous business.

Adjournment.

Time of session 10 A. M. until 2 P. M., and 7½ P. M. until adjournment.

Dr. Freeman, of Buffalo, moved that instead of an evening session, an afternoon session be held from 4.30 to 8 P. M. Lost.

The report of the Committee was then adopted.

Under the head of "Reports of Officers and Special Committees" the committee on credentials reported progress.

The Secretary read a communication from Dr. J. J. Birge, of San Francisco, tendering his resignation as a member, because of his being so far removed from the Atlantic States.

On motion the resignation was accepted.

The Executive Committee, through Dr. Shepard, of Boston, reported that for absentees, they had substituted Dr. G. V. Black, of Jacksonville, Ill., on the Committee on Appliances, and Dr. C. N. Pierce, of Philadelphia, on that of Nominations.

Dr. G. H. Cushing, of Chicago, moved the adoption of the following resolution offered at the last meeting:

*Resolved*, That Dentists not resident in the United States who may be present, and whose names may be approved by the Executive Committee, are hereby invited to seats upon the floor, and to participate in the discussions.

The Corresponding Secretary, Dr. Marshall H. Webb, made the following report:

Your Corresponding Secretary would respectfully report that he has attended to the duties assigned him, notified the gentlemen who were elected honorary members of the Association, viz: John Tomes, M. R. C. S.; Charles S. Tomes, M. A., M. R. C. S.; George Van Langadorff, D. D. S.; E. Magitot, M. D.; Adolph Petermann, D. D. S.; Mordant Stevens, M. D., D. D. S., M. R. C. S.; and Mr. Wedl.

A reply has been received from each of the gentlemen named excepting John Tomes.

In compliance with the resolution passed at the last meeting of the Association, your Corresponding Secretary, in connection with Dr. McQuillen, has seen to the getting up of a certificate of membership, a copy of which is here presented.

MARSHALL H. WEBB,
*Cor. Sec.*

Dr. Cushing moved the adoption of the following amendment to Article III. Section 4, offered at the last meeting:

All delegates shall be practitioners of dentistry; they shall receive their appointment only from permanently organized State Dental Societies and Dental Colleges.

On motion action on this amendment was indefinitely postponed.

A further amendment to Section 1 of Article III, "that each local society may send one delegate for every fifteen of its active members," was voted upon and lost.

Dr. R. F. Hunt, of Washington, D. C., presented the following majority report of the Special Committees on Sections, which was not adopted, a minority report prevailing:

*To the American Dental Association:*

The action of the American Dental Association in adopting the following resolution has been interpreted by the enlarged committee as a desire that the subject should be more maturely considered, and the results of that consideration laid before the Association in a more exhaustive report and a more detailed and perfected plan:

"*Resolved*, That the committee be increased to ten members, and that the report presented by the committee of three be recommitted to said committee for amendment or reconsideration, and that they be instructed to print the results of their deliberations, and that a copy be mailed to each member of the Association at least one month before the next annual meeting."

Acting upon this interpretation the committee will endeavor to set forth the advantages and objections attending the introduction of this very material change in the working machinery of the Association. On both these points it will be necessary to compare the probable working and results of the proposed system of permanent sections, with the known and tried ones of the present plan of annual committees. The committee cannot do better in that direction than to quote here the second and third paragraphs of the original report, as follows:

"The principle of division of labor and concentration of effort, so extensively adopted in the successful prosecution of ends in so many business interests, and that have been so happily practicalized in this body, would seem to apply with peculiar force to our investigations and researches, since we have before us a field so wide, and embracing so many branches of science that no one man can hope to master them all, or even a considerable number of them and at the same time pursue the practice of his profession.

"It can, therefore, hardly be doubted that the members of this Association can accomplish more for the advancement of the interests of our profession by persistently pursuing scientific inquiries, as connected therewith, in a single direction or on a limited number of subjects, than by the present system of preparing papers or essays year after year, and each year on different subjects."

This general comparison may be carried more into detail by saying that, by the plan of annual committees, members are appointed thereon in many instances without having been consulted, and thus placed in charge of subjects not consonant with their tastes or inclinations and with which they are frequently not familiar.

The papers prepared by the committees thus constituted, although of great value in themselves as embodying the views and opinions of prominent gentlemen of our profession, necessarily contain much of repetition and lose much of force and efficiency by reason of the unavoidable want of system in the treatment and presentation of the various subjects, and the equally unavoidable aspect which they bear of being but the expression of individual views and opinions.

It is believed that in the proposed system of permanent sections, if properly organized and operated, will be found a remedy for this and other objections. Each member of the Association will be free to select for himself that channel of study and labor which his acquirements, his present knowledge, his taste and predilections or his desire for special instructions may lead him to prefer. Each section, being permanent in its membership and continuous from year to year, can present to the Association the results of its labors so systematized and well digested as to be most effective, and the talent and ability of those members who will naturally gravitate to each section will, if not immediately, yet in a short time, give to its dictum on the subject under its charge, that type of authority that is now accorded to the writings of Agassiz on Natural History, Leibig on Chemistry, Huxley, Leidy and Marsh on Comparative Anatomy and Physiology, Tyndall on Physics, etc.

While each section is pursuing the investigation of its particular subject in a thorough and exhaustive manner in a scientific point of view, its members will in turn receive the benefits of similar labors of each of the other sections, thus making the results of the special researches of each section and individual enure more effectively to the benefit of the whole Association and the profession at large.

The objections to the adoption of the system of permanent sections would seem to lie, first, in the difficulty always attending the introduction and organization of new machinery; and second, in the doubt that may arise whether the members, after uniting with a section, would work so as to make its labors effective. These objections, however, would derive all their weight and force from a want of disposition on the part of the members, and not from any inherent defect or disadvantage of the system itself. Should it present itself in so favorable an aspect as to lead to its adoption, there would be certainly little difficulty, after its details are determined, in carrying it into practical operation. This would simply require willing, intelligent, and properly directed effort.

The first step would be the formation of the sections, which is accomplished by each member choosing his subject or special field of labor, and enrolling his name in the section having charge of it. The next would be the organization of the sections by the election of a chairman, and if desired, other officers of each section.

Upon this chairman, as its quasi executive officer, would naturally devolve the duty of conducting and supervising the work of the section—a trust which it is necessary in all large or small organizations to repose in some one—and this duty and trust always carries with it a greater or less amount of labor. The method of investigation and preparation of the work of the sections for report to the Association must, from the diversity of subjects and manner of handling them, be left to a great extent to the judgment of each section, although there should be a general similarity of plan for all.

It is here suggested that the chairman of each section divide his subject into as many parts or heads as a proper analysis or dissection will permit, and by correspondence or otherwise obtain the conclusions of each member of his section upon all or any one or more of these parts, and embody these conclusions with his own in the report prepared by him for the Association. Of course these conclusions should be based on careful investigation and supported by facts, because no proposition or statement has any value in a scientific point of view unless so based and supported; and one great objection in the introduction and adoption of this system, is to bring the diverse views, theories and propositions which are at this time, or may in future, come before our profession, to that test, and thus establish and fortify the true, and eliminate those having no solid foundation.

It would probably be well for this Association to constitute the chairmen of the several sections a committee to mature and adopt some general plan, and, as far as they can the details of the work of the section.

While it would be desirable that all the members of the Association should have the benefit of the discussions elicited in the sections while making up the reports, yet that is impossible. As now, the consideration of the report made would afford the best occasion for profitable discussion and instruction.

It will be readily understood that while the treatment of the past history and former developments of a subject may make the first two or three reports of a section lengthy, yet, when they shall have been disposed of, the current condition of such subject will probably furnish matter for only brief reports, and at that time, therefore, the number of divisions and sections reported in the following resolutions will not be too great, though at present it may seem cumbersome:

*Resolved*, That the membership of this Association be, and is hereby divided into twelve different sections, each section to be composed of such members as may elect to unite and form that section.

*Resolved*, That it shall be the duty of each member of this Association to connect himself with some one of these sections, but that he shall be free to follow his own preferences as to the particular channel in which he will labor, and may, if he so desires, be a member of two or more sections.

*Resolved*, That the members of each section shall at each annual meeting of the Association, immediately after the regular election of officers, elect in turn and in the order named, a chairman and such other officers as they may see fit, the names of the members of each section being read and the same tellers canvassing the ballot. And it shall be the duty of the chairman so elected to see that the labors of his section are conducted in the manner calculated to secure the best results.

*Resolved*, That each section shall have charge of one of the following divisions of the subjects or branches essential to scientific dentistry, and through its chairman, or such other member as it may select, report upon the same to the Association at each annual meeting, in the order in which they are named:

1. Operative Dentistry or Dental Surgery. 2. Artificial Dentistry. 3. Anatomy and Physiology. 4. Histology and Microscopy. 5. Etiology. 6. Pathology and Therapeutics. 7. Chemistry and Materia Medica. 8. Metallurgy and Chemistry of the Metals. 9. Dental Education. 10. Dental Literature. 11. Dental Nomenclature. 12. Dental Appliances.

*Resolved*, That after the several sections shall have been fully organized and in working condition, it shall be the duty of each chairman to furnish the latest and best information in the possession of his section upon any point connected with its subject, to any member of another section, who, through the chairman of his own section, may apply to him for such information.

Respectfully submitted,

R. Finley Hunt,
W. H. Atkinson,
H. A. Smith.
C. W. Spalding,
Homer Judd,
W. H. Morgan,
J. H. McQuillen,
*Committee.*

Dr. W. H. Atkinson, of New York, from the committee on Dental Nomenclature and Terminology, presented a report giving a few illustrations, suggestions, etc., for the improvement of technical terms in use in the profession. The committee recommended that the name of section two be changed from Dental Nomenclature to Nomenclatium, and the correlation of dentistry with science at large.

Discussion being in order, remarks on this subject were made by Dr. John Allen, of New York, who proposed the term "Artificial Dentistry" as a substitute for " Mechanical Dentistry."

The recommendation from the committee on nomenclature was laid on the table, until the report of the committee on sections came up for disposition.

After further remarks from Dr. J. Taft, of Cincinnati, O., the subject of nomenclature was passed.

Dr. Thomas Fillebrown moved that all dentists and physicians, not members of the Association, be invited to seats upon the floor. Carried.

The following proposed amendment to section 4, article III, of the constitution, offered by Dr. D. C. Hauxhurst, of Battle Creek, Mich., a year ago, and laid over, was, by request of Dr. J. H. McQuillen, of Philadelphia, taken up for action :

No Dental College shall be eligible to representation in this Association which does not require two courses of lectures as a condition of graduation.

Dr. Fillebrown moved its adoption.

Dr. Hunt earnestly opposed this motion, and the amendment.

Dr. A. O. Rawls, of Lexington, Ky., moved that action be postponed until the subject of dental education was brought up for consideration.

After remarks by Dr. H. A. Smith, of Cincinnati, Dr. Sheppard and Dr. C. W. Spaulding, of St. Louis, Dr. Klump, of Williamsport, rose to a point of order, claiming that debate was not in order.

The chair decided the point well taken under the rules.

Dr. W. H. Morgan, of Nashville, Tenn., briefly addressed the convention, and the question being taken on the motion of Dr. Rawls, it was lost.

The subject was discussed by Drs. Atkinson, I. J. Weatherby, of Boston, McQuillen, Spalding, J. Foster Flagg, C.

N. Pierce, T. L. Buckingham, Hunt, Morgan, Frank Abbott, G. B. McDonough, Rawls and others.

A majority of these gentlemen expressed themselves as conscientiously opposed to stipulating a stated time for graduation as it was arbitrary. They favored the adoption of a high standard and a course of examination, which should entitle an applicant to the degree, provided he passed a perfect examination and proved that he was equal to the grade of proficiency demanded.

Dr. George H. Cushing, of Chicago, moved that the subject matter be committed to a committee of three. Carried.

The Chair appointed as said committee Drs. Morgan, Fillebrown and Black. This Committee reported on Friday.

At two o'clock the Convention adjourned until evening.

The following is a list of the officers and standing committees:

*President.*—F. H. Rehwinkle.

*First Vice President.*—L. D. Shepard.

*Second Vice President.*—George T. Barker.

*Corresponding Secretary.*—M. H. Webb.

*Recording Secretary.*—M. S. Dean.

*Treasurer.*—W. H. Goddard.

*Executive Committee.*—W. H. Morgan, Chairman. M. H. Webb, Secretary.

*Committee on Appliances.*—Thomas Fillebrown, Chairman, A. L. Northrup, T. L. Buckingham.

*Committee on Credentials.*—L. D. Shepard, Chairman, M. H. Webb, J. N. Crouse.

*Committee on Nominations.*—Homer Judd, Chairman, W. H. Morgan, G. H. Cushing.

Among the most prominent in the dental profession who were in attendance upon the Association were Dr. W. H. Atkinson, of New York, Prof. T. L. Buckingham, of the Pennsylvania Dental College, Philadelphia, Prof. McQuillen, of the Philadelphia Dental College, Prof. Darby, of the Dental Department of the University of Pennsylvania, Prof. J. Taft, of the Ohio Dental College, Cincinnati, Prof. J. J.

Weatherbee, of the Boston Dental College, Prof. L. D. Shepard, of the Dental Department of Harvard University, Prof. C. W. Spalding, of the Missouri Dental College, Dr. R. Finley Hunt, Washington, D. C., Prof. Frank Abbott, New York College of Dentistry and Prof. F. J. S. Gorgas, of the Baltimore College of Dental Surgery.

An elaborate display of dental appliances and laboratory apparatus was made by the Buffalo Dental Manufacturing Company in charge of Dr. T. G. Lewis; S. S. White of Philadelphia, in addition to instruments, exhibited a full line of operating chairs and material; Johnston Brothers of New York had also a fine display of goods of like character, the principal articles being the Wilkerson Dental Chair and a Dental Engine. R. S. Williams of New York exhibited a variety of gold foil, etc. Dr. Bonnwell, of Philadelphia, also displayed a Dental Engine and Chair.

TUESDAY.—Evening Session.

The meeting of the American Dental Association was continued at eight o'clock, Tuesday evening, President Rehwinkle in the chair.

On motion the rules were suspended for the transaction of miscellaneous business.

Report of the Treasurer.—

| | |
|---|---|
| Balance on hand 1877, - - - - | $288 64 |
| Cash received for dues, - - - - | 770 00 |
| | $1,058 64 |
| Disbursements, - - - - - | $899 95 |
| Balance on hand, - - - - - | $158 69 |

On motion the chair was instructed to appoint two members to fill vacancies on the committee for prize essays. Drs. Pierce and Morgan were accordingly named.

Dr. Shepard presented the following minority report from the committee on sections:

Minority Report.—The undersigned members of the Committee on Sections, did not sign the report with the majority, for various reasons:

They were and are in doubt as to the wisdom of dividing the Association into sections, but as the opinion of the members in favor seems to predominate, they would recommend that the experiment be tried. They would also recommend that at first the sections should be few in number, and then sub-divided as may seem desirable. As a resolution is of no effect in opposition to the Constitution, they beg now to present, without further argument, a minority report in the following draft of amendments to the Constitution:

ARTICLE III.—*Sec.* 3. Insert the words "and membership of the sections," between the words "to office," at the end of the sixth line, and before the words "none being eligible," at the commencement of the seventh line.

ARTICLE IV.—*Sec.* 6. Strike out the sentence "they shall also report to the Association upon any and all appliances which may be placed in their hands," near the bottom of the seventh page of the printed Constitution.

ARTICLE V.—*Sec.* 6. Page 8. Under head of "third division" strike out the words "names for standing Committees for the ensuing year and" between the words "they shall report," and "the names of places."

ARTICLE VI.—Strike out all five Sections of Article VI, and insert a new article as follows:

*Sec.* 1. To prepare, arrange and expedite business, this Association shall be divided into six sections as follows:

1. Artificial Dentistry, Metallurgy and Chemistry. 2. Dental Education. 3. Dental Literature and Nomenclature. 4. Operative Dentistry. 5. Anatomy, Physiology, Histology, Microscopy and Etiology. 6. Pathology, Therapeutics and Materia Medica.

*Sec.* 2. It shall be the duty of each permanent member to inform the Recording Secretary, before the close of the morning session of the third day of the annual meeting, which of the six sections he elects to join for the ensuing year. He shall be free to follow his own preferences as to the particular channel in which he will labor, but can be a member of but one section during the same year.

*Sec.* 3. It shall be the duty of the Recording Secretary immediately after the close of the above morning session of the third day, to make out lists of the names thus furnished of each section, as a basis for the election of officers of said sections.

*Sec.* 4. The Association shall, by vote, assign some time after the regular election of officers of the Association, for the meeting of the several sections for organization.

*Sec.* 5. When the time for the organization of the sections has arrived, it shall be the duty of the Recording Secretary to furnish some one member of each section with the list of members of his respective section, which member shall thereupon call the members to order, and read the list of names. The members shall then elect by ballot, a chairman and such other officers as they may see fit.

*Sec.* 6. The chairman shall preside at all the meetings of his section. Shall exercise general supervision over the business, and shall see that the labors of his section are conducted in the manner calculated to secure the best results. The chairman, or such other member or members as the section shall select, shall prepare and read at the annual meeting of the Association next ensuing, a paper or papers on the advances and discoveries of the past year in the branches included in the section. The reading of such papers shall not together occupy more than forty minutes.

*Sec.* 7. The several sections shall meet at 10 A. M. on the morning of the first day of the annual meeting of the Association.

*Sec.* 8. For the information of all members, a list of the members of each section shall be published in the transactions of each year.

Article VIII.—Under "order of business" insert between Nos. 1 and 2 the words 2 meetings of the sections at 10 A. M. Change present No. 2 to 3, and have it read " Organization of the Association at 11 A. M. Change No. 3 to 4. Change No. 4 to 5, and strike out the words " standing committees," and insert the word "sections." Change Nos. 6, 7, and 8 to 7, 8, and 9. Strike out all of No. 9.

Respectfully submitted,

L. D. SHEPARD.
J. N. CROUSE.

When he had concluded reading the report Dr. Fillebrown moved that it be accepted, and to lay on the table the majority report. Carried.

DENTAL PHYSIOLOGY.—Dr. Dean read his report on dental physiology, which, while it did not present any new developments in relation to the subject, gave a sufficiency of matter for discussion and elaboration. It first dealt with the rudimentary germs of teeth and gave the conclusions of certain investigators who have sought for the presence of those germs in fœtal substances. He took exception to the claims of Goodsir, on the ground that they need greater verification, or must be abandoned altogether. The next point was the consideration of the third and fourth dentations, and the idea of reproenacy of this kind in old age was scouted by the author. While making mention also of the tendency of scientific men to be led in to discrepancies regarding the teeth. With reference to the opinion of Dr. Magitot, that the crown of the tooth is governed by the enamel organ and not the dental papilla as is generally supposed. The belief of Dr. Dean had been strengthened that the enamel organ is the most passive structure and is formed by the dental papilla. This question was elaborated to considerable extent and in a manner highly interesting to the members of the Association.

When Dr. Dean had concluded, his paper being open for discussion, Dr. Atkinson took the floor and said the able and most excellent paper presented, properly belonged to histology. He then made a very scientific and instructive address upon the subject brought up. He gave notice that it was his intention to place before the convention before adjournment certain drawings, etc., which would demonstrate conclusively to the eyes and ears of the members what it is that gives form and direction to the teeth.

Dr. J. H. M'Quillen next read a paper on Physiology, in which he gave an account of the restoration of certain functions or voluntary action of which a pigeon had been deprived by the removal of four-fifths of the upper portion of the cerebrum, while another had died from the effects produced by a removal of the cerebellum.

We present the following interesting abstract from this paper:

"The usual phenomena attendant upon the operation followed, in profound stupor, the bird standing motionless on the table, with eyes closed, the head sunk between the shoulders and the feathers ruffled. When pushed, it opened its eyes and moved the body, and when thrown into the air it flew a few feet and then, on alighting, relapsed into somnolency with an evident obliviousness to surrounding objects until again aroused by handling. By the first of March, 1878, about twenty-three days after the operation, I was surprised at the complete recovery of the voluntary movements of walking and flying, the general manifestation of intelligence and the fact of feeding itself and drinking as usual. I asked then and I repeat now the same question: how are we to account for the restoration of these functions? Is it due to the fact that the small amount of cerebrum left after the operation assumed the functions of the entire organ, or has there been a regeneration of the part removed? There is but one other case on record that I have met with, where there has been a recovery of voluntary action on the part of a pigeon from which the cerebral hemphiseres had been removed; and I was not aware of that fact until the experience with my pigeon induced me to make a careful examination of the literature of the subject. It must be remembered I only cut out the upper four-fifths of the cerebrum. In doing this, however, the superficial gray matter of the hemphiseres, reorganized generally as the structure physiologically concerned in the exercise of the faculties of attention, perception, memory and will, was removed."

When he had concluded reading his paper, Dr. Mc Quillen said that six months after the operation the pigeon which survived the vivi section was subsequently put to death under chloroform and a *post mortem* examination made. On removing the scalp a fibrous structure was found analogous to the pericranium and on opening this a small amount of serous fluid escaped. When the cranial cavity was exposed he found a large amount of a white substance which he inferred was the regenerative of the cerebrum removed. He placed a section of it under the microscope and discovered that it held ley-polar cellars characteristic of nervous structure. There were those who opposed vivisection, saying " we have learned all we can, don't torture animals any more. Dr. McQuillen thought his experience had taught him that there was yet much to learn in order that dentists as the alleviator's of human suffering might appreciate what was expected of them and be stimulated to govern themselves accordingly.

We can have no better evidence, said the speaker, of the necessity, of experimental investigation in the nervous system, than was presented by the statement of Prof. Brown-Sicord, the distinguished scientist, who has succeeded to the chair held by Claude Bernard in Paris, that most of the conclusions of physiologists and pathologists, in this direction, are founded in error. As he has said, the superstructure is not built upon the rock but on the sand, and that it is tumbling about our ears. When Dr. McQuillen had concluded, he was greeted with a storm of applause.

Association then adjourned until Wednesday morning.

### WEDNESDAY.—Morning Session.

The second morning session of the annual meeting of the Association was opened at ten o'clock in the usual form, Dr. F. H. Rehwinkle presiding.

The report of the Committee on Histology and Microscopy was called for, and the chairman, Dr. G. V. Black, read a very interesting paper on the " Odonto-blasts," the leading features of which were illustrated with and made doubly interesting by microscopial diagrams and blackboard drawings.

Discussions being in order, Dr. Spalding took occasion to highly compliment the paper of Dr Black. No further remarks being offered by members, Dr. G. F. Waters, of Boston, from the same committee, was called upon to present the next paper. He addressed the convention *ex-tempore* upon "Fungi in the Mouth," giving the results of his investigations with reference to the subject he had chosen. His remarks were accompained with blackboard illustrations of what he had found in the way of fungus growth upon the surface of a tooth.

Dr. Waters was followed by Dr. C. N. Pierce, who read a special paper upon the mechanical genesis of tooth form, showing that force employed by the lower jaw has had its influence in modifying tooth forms.

At the conclusion of his paper, Dr. Pierce presented the general report of the Committee on Histology and Microscopy, which embraces the important announcement that W. R. Gowans, of London, England, had perfected an instrument for the counting of blood corpuscles, whereby he finds that the disproportion of white and red corpuscles is greater than was supposed. The use of iron, he had also found, greatly increases the number of red corpuscles, even to an enormous extent, without improving the health. This augmentation of the red globules was useless without a corresponding amount of hemi-globine. The report concluded with a reference to the importance and need of dentists receiving more systematic and thorough training outside of their manipulative work. It was the knowledge which would fit them to deal with those stubborn anæmic cases which are so productive of *Neuritis*.

Dr. W. H. Atkinson next read a valuable scientific essay, written by Dr. C. F. W. Bœdecker, D. D. S., of New York, a non-member of the Association, the subject of which was the preparation and treatment of specimens of the teeth and gums for the microscope. It showed considerable research upon the part of the author and brought forward a number of new views upon heretofore disputed questions.

Prof. T. L. Buckingham discussed the formation of dentine. The foundation of all organized tissue, he said, was the cell, from which comes another cell similar to the first, the same as an animal from an animal, etc. In other words it was a system of reproduction, the cells multiplying very rapidly, in some instances a cell growing in two or three minutes. In time the germinal matter becomes hardened and forms material, while the cells it has produced go on producting. An aggregation of cells will sometimes produce a dissimilar cell. In the tooth the germinal matter is pushed in or down and the material or dentine is left behind.

Dr. Atkinson followed Prof. Buckingham, and said that those who were depending upon the views of authors whose books and writings are ten years old, were wasting their time. Cells, he said, are vegetable products and there is no such thing as a cell in the human body except so far as they are of vegetated growth.

At the conclusion of the doctor's remarks, he moved that an afternoon session be held from four until six o'clock, to expedite business.

Dr. Spaulding moved as an amendment that the extra session be held Thursday afternoon. Carried.

The convention then adjourned until evening.

[TO BE CONTINUED.]

---

